# Mapping Psychosocial Disability in Schizophrenia: Network Analysis of a Relatives-Rated Clinical Scale

**DOI:** 10.1192/j.eurpsy.2026.10236

**Published:** 2026-07-31

**Authors:** S. F. Frileux, N. Vidal, E. Brunet, P. Roux

**Affiliations:** 1 UVSQ, Montigny le Bretonneux; 2 CESP, Villejuif; 3 Versailles Hospital, Le Chesnay, France

## Abstract

**Introduction:**

Schizophrenia is marked by major impairments in psychosocial functioning. Disability arises from intertwined processes, notably impairments in cognition, motivation, social interactions, and metacognition. The Evaluation of Cognitive Processes involved in Disability in Schizophrenia (ECPDS) is a 13-item scale completed by relatives, focusing on these four processes in daily life. Network analysis provides a framework to examine their interconnections, identify central nodes as potential intervention targets and explore links with external factors.

**Objectives:**

We tested whether clustering from a network analysis of the ECPDS provides insights beyond its established four-factor structure, identifies items associated with neurocognition, global functioning, quality of life and anticholinergic burden, in order to define intervention targets to guide innovative approaches for psychosocial disability in schizophrenia.

**Methods:**

Data came from the FondaMental FACE-SZ cohort, including patients with schizophrenia spectrum disorders; after exclusions, analyses focused on 429. Networks were estimated as regularized partial correlation networks (EBICglasso), with edge and centrality stability assessed via bootstrapping. External variables were a global neurocognition score, the Personal and Social Performance scale (PSP, a clinician-rated measure of overall psychosocial functioning in domains conceptually distinct from the ECPDS, which captures relatives’ perspectives on underlying cognitive and motivational processes), the Subjective Quality of Life scale (SQOL), and the Salahudeen anticholinergic burden scale.

**Results:**

Three clusters emerged in the ECPDS network (image 1): (1) **Cognitive Impairments**, (2) **Avolition**, (3) **Socio-Metacognitive Deficits**. Strongest ECPDS nodes were “Routine organization”, “Adaptation”, “Curiosity”, and “Perspective-taking”. Neurocognition was linked with “Routine organization”, “Non-routine organization”, and “Learning”. PSP connected with “Initiative”, “Project”, and “Use of time” (image 2). For SQOL, only “Use of time” showed a reliable association(image 3). Salahudeen scale related to “Organization”, “Adaptation”, “Learning”, and “Attention”. Bootstrap analyses confirmed stability of centrality estimates.

**Image 1:**
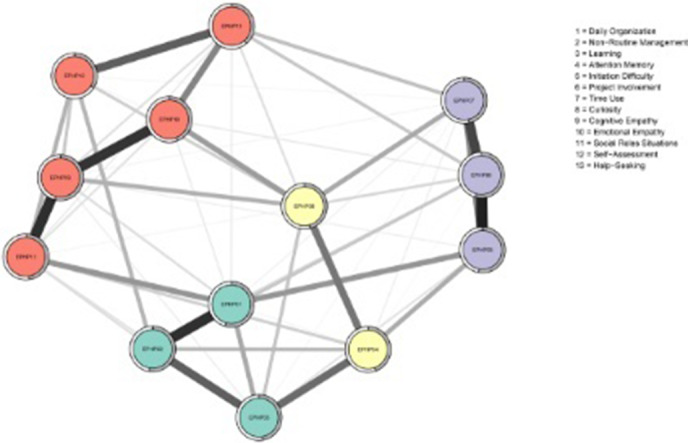
Image 1: Long description.

**Image 2:**
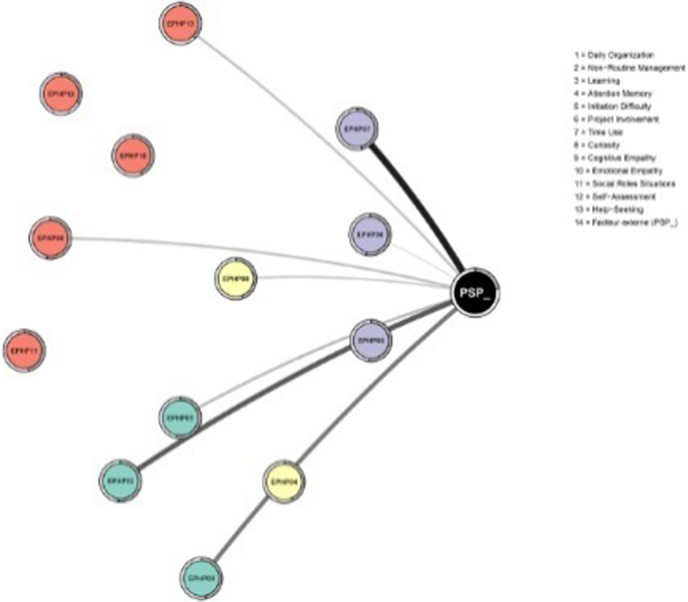
Image 2: Long description.

**Image 3:**
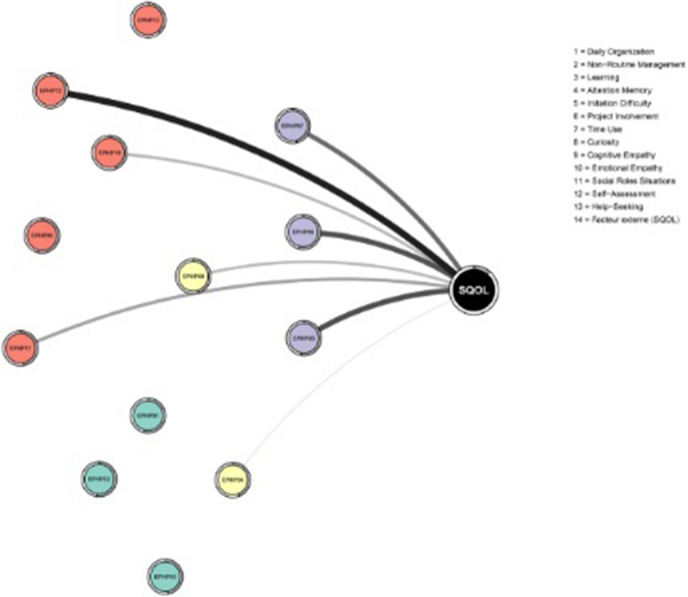
Image 3: Long description.

**Conclusions:**

Relatives’ assessments with the ECPDS provide specific insights into psychosocial disability. Difficulties in “organization” and “learning”, when reported by relatives, may help detect patients needing extended neuropsychological assessment. ECPDS scores may also reflect treatment effects, improving with adequate dosing but impaired by anticholinergic burden. Neurocognition, psychosocial functioning and quality of life each captured different aspects, underlining the composite nature of the ECPDS and its potential to guide targeted interventions, with consequences for cognition, functioning, and quality of life.

**Disclosure of Interest:**

None Declared

## Long descriptions

### Image 1: Long description

A network diagram with 13 circular nodes connected by lines of varying thickness and opacity.

At the center is a yellow node labeled E P I P 0 8.

In the Northwest quadrant, a cluster of five red nodes includes E P I P 1 1 at the top, E P I P 0 2 and E P I P 1 0 below it, and E P I P 0 9 and E P I P 0 1 at the far left. These are connected by thick black lines.

In the Northeast quadrant, a cluster of three purple nodes includes E P I P 0 7, E P I P 0 6, and E P I P 0 5, connected vertically by thick black lines.

In the Southwest quadrant, two teal nodes labeled E P I P 1 2 and E P I P 1 3 are connected to each other and the central node.

At the South, two yellow nodes labeled E P I P 0 4 and E P I P 0 3 are connected to the teal cluster and the central node.

A legend on the right lists the following numbered items.

1 equals Daily Organization.

2 equals Non-Routine Management.

3 equals Learning.

4 equals Attention Memory.

5 equals Initiation Difficulty.

6 equals Project Involvement.

7 equals Time Use.

8 equals Curiosity.

9 equals Cognitive Empathy.

10 equals Emotional Empathy.

11 equals Social Roles Situations.

12 equals Self-Assessment.

13 equals Help-Seeking.

Navigate back to Image 1:.

### Image 2: Long description

A network diagram features a central black node labeled P S P underscore on the right side. Radiating from this hub are several lines of varying thickness connecting to colored circular nodes.

Connections from the P S P hub include:

- A thick black line to a purple node labeled E P H P 0 7.

- A thin gray line to a purple node labeled E P H P 0 6.

- A thin gray line to a yellow node labeled E P H P 0 8.

- A medium gray line to a purple node labeled E P H P 0 5.

- A thick gray line to a teal node labeled E P H P 0 1.

- A thin gray line to a yellow node labeled E P H P 0 4.

Secondary connections include:

- E P H P 0 1 connects to teal node E P H P 0 2.

- E P H P 0 4 connects to teal node E P H P 0 3.

- E P H P 0 8 connects via a very faint line to red node E P H P 0 9.

- E P H P 0 7 connects via a very faint line to red node E P H P 1 0.

Isolated red nodes on the far left include E P H P 1 2, E P H P 1 3, and E P H P 1 1.

A legend on the right lists 14 items:

1 equals Daily Organization.

2 equals Non-Routine Management.

3 equals Learning.

4 equals Attention Memory.

5 equals Initiation Difficulty.

6 equals Project Involvement.

7 equals Time Use.

8 equals Curiosity.

9 equals Cognitive Empathy.

10 equals Emotional Empathy.

11 equals Social Roles Situations.

12 equals Self-Assessment.

13 equals Help-Seeking.

14 equals Facteur externe (P S P underscore).

Navigate back to Image 2:.

### Image 3: Long description

A network diagram features a central black node labeled S Q O L on the right side. Multiple curved lines of varying thickness connect this hub to a cluster of colored nodes on the left.

From top to bottom, the connected nodes include:

* E P H P 1 3 (red) connected by a thick black line.

* E P H P 0 7 (purple) connected by a medium grey line.

* E P H P 1 0 (red) connected by a thin grey line.

* E P H P 0 4 (purple) connected by a thick dark grey line.

* E P H P 0 8 (yellow) connected by a thin grey line.

* E P H P 0 5 (purple) connected by a thick dark grey line.

* E P H P 1 1 (red) connected by a thin grey line.

* E P H P 0 6 (yellow) connected by a very faint thin line.

Several nodes are disconnected in the bottom left, including E P H P 1 2 (red), E P H P 0 9 (red), E P H P 0 1 (teal), E P H P 0 2 (teal), and E P H P 0 3 (teal).

A legend on the far right lists 14 categories:

1 = Daily Organization

2 = Non-Routine Management

3 = Learning

4 = Attention Memory

5 = Initiation Difficulty

6 = Project Involvement

7 = Time Use

8 = Curiosity

9 = Cognitive Empathy

10 = Emotional Empathy

11 = Social Roles Situations

12 = Self-Assessment

13 = Help-Seeking

14 = Facteur externe (S Q O L)

Navigate back to Image 3:.

